# National Oral Health Policy and Financing and Dental Health Status in 19 Countries

**DOI:** 10.1016/j.identj.2023.01.007

**Published:** 2023-03-21

**Authors:** Tess Foote, Lauren Willis, Tracy Kuo Lin

**Affiliations:** a School of Dentistry, University of California, San Francisco, California, USA; b School of Dentistry, Columbia University, New York, New York, USA; c Institute for Health & Aging, Department of Social and Behavioral Sciences, School of Nursing, University of California, San Francisco, California, USA

**Keywords:** Oral health policy, Financing, Oral health, Global public health, DMFT

## Abstract

**Objective:**

Dental caries in permanent teeth is one of the most common health issues—despite being preventable in early stages—due to inadequate regulation of preventive dental services in many countries. This study evaluates the association between regulation of preventive dental services and oral health outcomes.

**Methods:**

This mixed-method study analysed data from 19 member countries of the Organisation for Economic Co-operation and Development (OECD). Oral health outcomes were measured using decayed missing and filled teeth (DMFT) indexes for children aged 12 to 18 years. Oral health expenditures were measured as a percentage of each country's gross domestic product (GDP). We conducted web-based research and systematically extracted and coded data on dental policy regarding children's preventive dental services. Preventive care was assessed based on legal policy mandating children receive preventive services, availability of free services for children, and regulation of the services provided. We assessed the relationship amongst oral health policy, outcomes, and expenditure using bivariate regression analysis.

**Results:**

The most common preventive policy category is the availability of free dental services for children (78.95%), and the least common is policy mandating dental services for children (26.32%). The oral health expenditure is correlated with DMFT index (−4.42, *P* < 0.05). The legal policy mandating dental services for children is correlated with DMFT index (−1.32, *P* < 0.05) and correlated with average oral health expenditure (0.16, *P* < 0.05).

**Conclusions:**

A percentage increase in oral health expenditure is associated with a 4.42 reduction in DMFT. The existence of legal policy mandating dental care for children is associated with a 1.32 reduction in mean DMFT score and a 0.16% increase in oral health expenditure. These findings highlight the importance of preventive care and may aid policymaking and health system reforms.

## Introduction

Dental caries in permanent teeth is one of the most common global health issues despite being preventable and treatable in early stages.^1^^,^^2^ The high prevalence of caries is partly because many dental services have often been dismissed as a nonessential component of health systems. Consequently, these services are excluded from the global movement towards universal health coverage in most countries.^1^^,^^3^

Ample evidence supports the positive effect of preventive measures on improving oral health outcomes. Preventive oral health services—such as dental screenings, counseling, and application of topical fluoride—reduce kindergarteners’ decayed, missing, and filled teeth (DMFT) indexes in the United States.^4^ Policy reform to increase the coverage for dental sealants in children resulted in a reduction in DMFT indexes in children in South Korea.^5^

Preventive dental measures reduce the progress of caries development and invasive treatments needed for more extensive decay and may be cost-effective and cost-saving. For example, Medicare beneficiaries who used preventive dental care had more dental visits but lower dental expenses than beneficiaries who sought treatment solely for problems.^6^ A cost-effectiveness analysis evaluated sealing all permanent molars (SA), sealing based on levels of risk (RBS), and sealing none (SN) and found that the RBS strategy improved clinical outcomes—in terms of cavity-free months—and saved money, when compared to the SN strategy. The SA strategy improved outcomes further but with additional costs.^7^

Given the evidence on the benefits of preventive dental care, health systems should promote disease prevention, allow for early detection, and provide suitable intervention.^8^ However, the absence of preventive dental care regulation combined with inadequate insurance coverage contributes to a low utilisation rate of preventive dental services in many countries.^9^ National and subnational regulation of the frequency and extent of preventive dental care should be associated with an improvement in dental outcomes. Some studies have examined this relationship between regulation and dental outcomes in the adult population.^9^, ^10^, ^11^ However, no quantitative dental policy analysis of paediatric oral health care has been conducted.

This mixed-methods study has 2 objectives: (1) to evaluate the roles of legal policy, access, and regulation in improving countries’ oral health systems and outcomes and (2) to assess the association between national oral health policies and guidelines—specifically for preventive care—and oral health expenditure and outcomes in selected member countries of the Organisation for Economic Co-operation and Development (OECD). This group of high-income and mostly democratic countries is equipped with the financial resources to provide well-rounded preventive oral health care. We hypothesize that countries with more regulations requiring preventive care are more likely to be associated with better oral health outcomes and lower oral health expenditures. Findings on the association between national oral health policies and outcomes will enable governments and policymakers to make evidence-based decisions when implementing health system reforms.^12^

## Methods

### Variables and data sources

#### DMFT indexes

We utilised countries’ DMFT indexes as proxy variables for oral health outcomes. DMFT is the sum of a person's decayed, missing, and filled permanent teeth. The DMFT index is a well-established measure of caries burden in oral epidemiology. A higher DMFT index indicates worse caries burden and further deterioration or oral health.^13^^,^^14^ We extracted DMFT data for children aged 12 to 18 years, allowing assessment of national regulations and guidelines which target preventive care for children up to the age of 18, from the WHO Oral Health Country/Area Profile Project (CAPP).^15^ See Appendix A for more details.

#### Oral health expenditure

We extracted oral health expenditure data from the OECD Health Expenditure and Financing database.^16^ The oral health expenditure considers all dental-sector curative care services provided in an outpatient setting,^17^ measured as a percentage of each country's gross domestic product (GDP). The OECD database characterises dental-sector curative care services as all services related to oral health, gums, and teeth and other related disorders; this group of services includes most dental services provided in an outpatient setting.^17^ Details of the extraction and missing observations can be found in Appendix B.

#### Oral Health Policy and Guidelines

Country-specific research was conducted for each of the OECD member countries, and countries were assessed based on their level of oral health prevention policy for children. All searches were first conducted in English and then in each country's native language to ensure that all the available data were being accessed; translations for search terms, websites, and documents were conducted using Google Translate. See Appendix C for search terms.

Evaluation of all the relevant policy and guideline documents indicated that some countries have legal policies mandating dental care for children, some have insurance schemes ensuring free services for children, and some have published clinical guidelines on providing preventive care. Therefore, we generated binary variables to indicate the availability of the following types of policies: *mandatory dental services for children; availability of free dental services for children;* and *available guidelines for children's preventive services*. The category *mandatory dental services for children* signifies whether a particular country has an existing law or act that specifically mandates children receive dental care including preventive services like oral health screenings, instruction, and fluoridation. This category indicates that a specific law indicating that the entire population should have access these services but does not measure the actual frequency of utilisation amongst the population. The category of *availability of free dental service for children* signifies whether a particular country's mandatory national insurance scheme or national public service includes preventive services for children in its basic dental coverage. The category of *available guidelines for children's preventive services* signifies whether a particular country's government or health ministry has published clinical practice guidelines for dentists on providing preventive care to children. This categorisation system allowed us to assess each country's level of regulation of children's dental services, predominantly preventive. Examples of these services are application of sealants, oral health screenings, fluoride varnish, and oral hygiene instruction. See Appendix C for additional details.

#### Countries

We focused on OECD member countries as these are high-income countries are more likley to have the resources to provide comprehensive dental care. The OECD works with governments and policymakers to establish evidence-based international standards^18^; we expected these countries to have standardised and accessible policy documents. A list of OECD countries excluded from the study due to insufficient public data can be found in Appendixes A and B. After accounting for these limitations, we included 19 of the 38 OECD member countries.

#### Limitations

When analysing oral health expenditure data, we focused on dental outpatient care and excluded dental inpatient procedures; the OECD database does not distinguish between dental inpatient services and nondental inpatient services. Whilst some costs may not be captured, most dental care—especially preventive care and caries treatment—are outpatient procedures.^17^

Additionally, health ministry websites often listed existing laws and regulations without indicating how these specific policies were enforced. This study therefore does not account for the frequency of utilisation of the services and only captures the fact that a country addresses the need for the services in its legislation. The policy data collection component of this study was conducted utilising Google Translate, and there is the possibility that certain text was not translated accurately. A more comprehensive database that monitors international oral health policy updates across languages is needed to allow for more detailed analysis.

This study averaged the DMFT indexes of children from the age of 12 to 18 years and assumed that caries experience does not change that significantly over this time period.^9^, ^19^ We reasoned that this age range is sufficiently narrow, encompassing only children within similar life stages and allowing for inclusion of additional data points for analysis. As caries experience does not develop steadily over one's lifetime, many argue that DMFT cannot be averaged for the entire population of a country. This argument stems from the significant differences in the caries experience of children and of an older population, especially because DMFT is an irreversible index.^20^ Nevertheless, despite its limitations, DMFT has been regarded as one of the most prominent indicators of caries experience.^13^, ^14^, ^21^

Both the OECD Health Expenditure and Financing and the WHO CAPP databases had missing values for certain years (detailed in Appendix B). Dental public health research particularly suffers from incomplete datasets, and more standardised data collection methods for oral health measures are needed globally. We recognise that our results may be biased towards countries that devoted more resources to dental care and country-level data collection. It is critical to underline that the trends may be different depending on a country's income level and regime type. We recognize that in most developed countries, dental caries has a skewed distribution, affecting vulnerable groups more severely.^12^ The within-country variation of DMFT is an important topic that is beyond scope of this study and should be further examined in the future; in this study, we focused our efforts on investigating country-level policy and DMFT variations. There are many confounding variables that affect the population-level DMFT index, such as socioeconomic status, race, and education level; these variables and their relationship with DMFT should be systematically examined when data become available.^12^

#### Statistical methods

The statistical software STATA version 17.0 was used to code, clean, and analyse the datasets.^22^ Descriptive statistics were generated for all indicator variables. We calculated bivariate regression analysis between each variable to examine the strength of associations between all indicator variables. Scatterplots were used to evaluate the trends amongst DMFT, oral health expenditure, and level of oral policy.

## Results

Table 1 summarises country characteristics with the mean (SD), minimum, maximum, and number of observations of average oral health expenditure and average DMFT as well as the data for each policy category, presenting the percentages of countries that implemented each policy. The least common policy implemented is *mandatory dental services for children* (26.32%), and the most common policy is *available dental services for children* (78.95%).

**Table 1:** Summary statistics for country characteristics and oral health policy categories.

Country characteristic	Mean (SD)	Min	Max	Observations
Average oral health expenditure as a % of GDP	0.45 (0.14)	0.20	0.80	19
Average DMFT index	1.83 (1.12)	0.50	5.11	19

**Policy category**	***Mandatory dental services for children***	***Availability of free dental services for children***	***Available guidelines for children's preventive services***	***Observations***

Yes	5 (26.32%)	15 (78.95%)	6 (31.58%)	26
No	14 (73.68%)	4 (21.05%)	13 (68.42%)	31

DMFT, decayed missing and filled teeth.

**Fig. 1:**
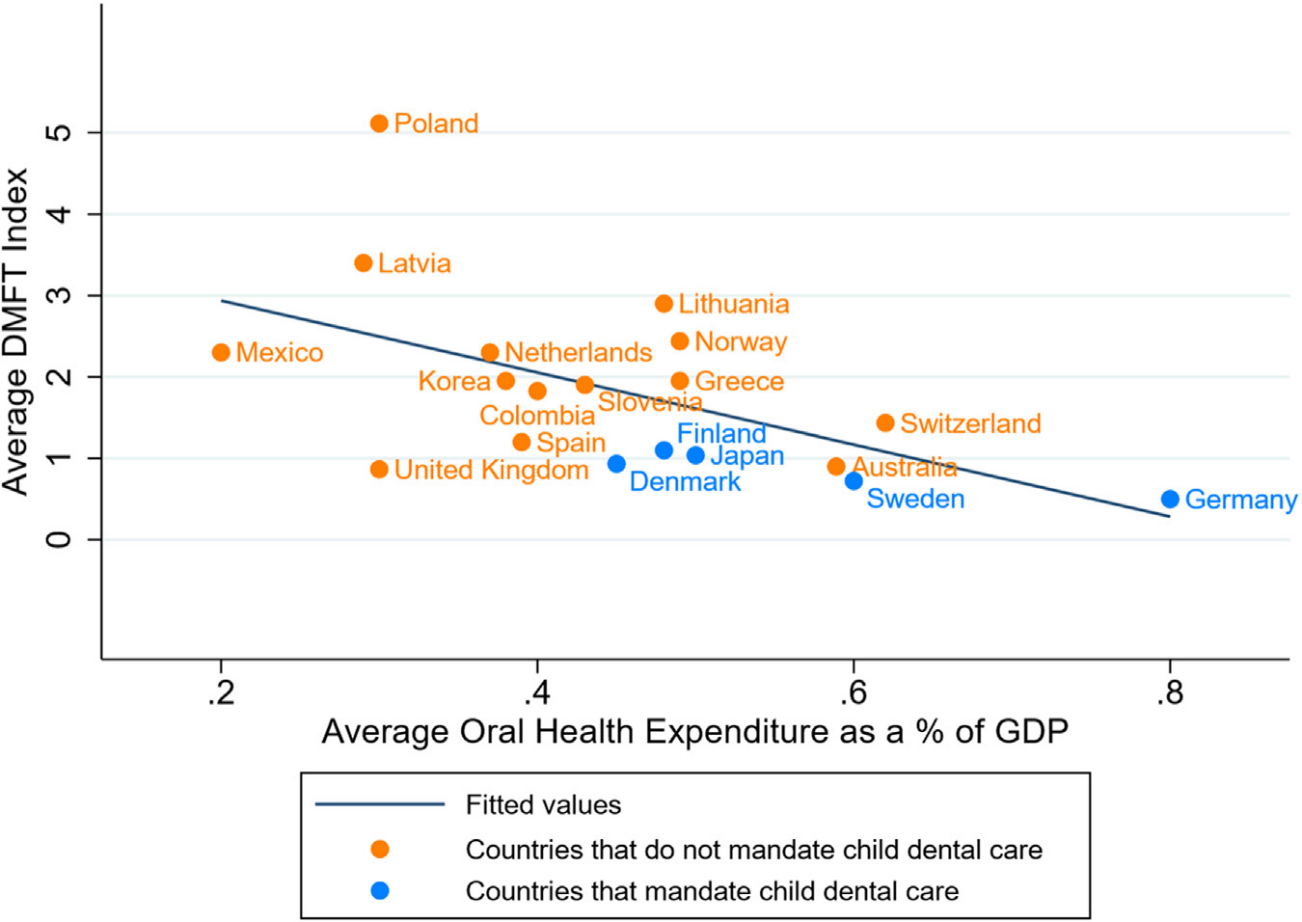
Scatterplot illustrating the average decayed missing and filled teeth (DMFT) index and average oral health expenditure as a percentage of GDP, colour-coded by legal policy.

Figure 1 is a scatterplot illustrating the relationship amongst average DMFT index, average oral health expenditure, and presence of a legal policy that mandates dental care for children. Countries with legal policies mandating dental care for children have lower DMFT indexes but slightly higher oral health expenditures. A sensitivity analysis was conducted; further information can be found in  Appendix D.

Table 2 presents information on policies implemented in each of the 19 countries included in this study. Denmark and Japan are the only 2 countries that not only mandate dental services for children but also have available dental services for children and guidelines for preventive dental care. Spain and Switzerland were the only 2 countries with no policies in any category. Mexico and Colombia have technical standards and guidelines, which were not commonly found in other countries. Most countries included in this study have available dental services for children, except Spain, Switzerland, Mexico, and Colombia.

**Table 2:** Policy categories by country.

Country	Mandatory dental services for children	Availability of free dental services for children	Available guidelines for children's preventive services
Australia	No	Yes	No
Colombia	No	No	Yes
Denmark	Yes	Yes	Yes
Finland	Yes	Yes	No
Germany	Yes	Yes	No
Greece	No	Yes	No
Japan	Yes	Yes	Yes
Korea	No	Yes	No
Latvia	No	Yes	No
Lithuania	No	Yes	No
Mexico	No	No	Yes
Netherlands	No	Yes	Yes
Norway	No	Yes	No
Poland	No	Yes	No
Slovenia	No	Yes	No
Spain	No	No	No
Sweden	Yes	Yes	No
Switzerland	No	No	No
United Kingdom	No	Yes	Yes

Table 3 presents findings on bivariate regression analysis. Oral health expenditure was negatively correlated with DMFT index (−4.42, *P* < 0.05), suggesting that a 1% increase in oral health expenditure is correlated with a 4.42 reduction in DMFT. This finding signifies a correlation of a child having approximately 4 fewer decayed teeth for each percentage point increase in spending. The average DMFT indexes of countries and regions vary significantly due to resource allocation and socioeconomic factors, but a decrease of 4 units in DMFT score would move most countries from a moderate to low DMFT.^23^ The legal policy for *mandatory dental services for children* was negatively correlated with DMFT index (−1.32, *P* < 0.05), suggesting that the existence of such a legal policy is correlated with a 1.32 reduction in mean DMFT score. The legal policy for *mandatory dental services for children* was also positively correlated with average oral health expenditure (0.16, *P* < 0.05), suggesting that the existence of such a legal policy is correlated with a 0.16% increase in oral health expenditure.

**Table 3:** Bivariate regression for associations between variables included in analysis.

	Average DMFT index	Average oral health expenditure as a % of GDP	Mandatory dental services for children	Availability of free dental services for children	Available guidelines for children's preventive services
Average DMFT index	1.00				
Average oral health expenditure as a % of GDP	−4.42*	1.00			
Mandatory dental services for children	−1.32*	0.16*	1.00		
Available dental services for children	0.18	0.06	0.33	1.00	
Technical standards/guidelines	−0.42	−0.12	0.10	−0.18	1.00

⁎ *P* < .05. DMFT, decayed missing and filled teeth.

**Fig. 2:**
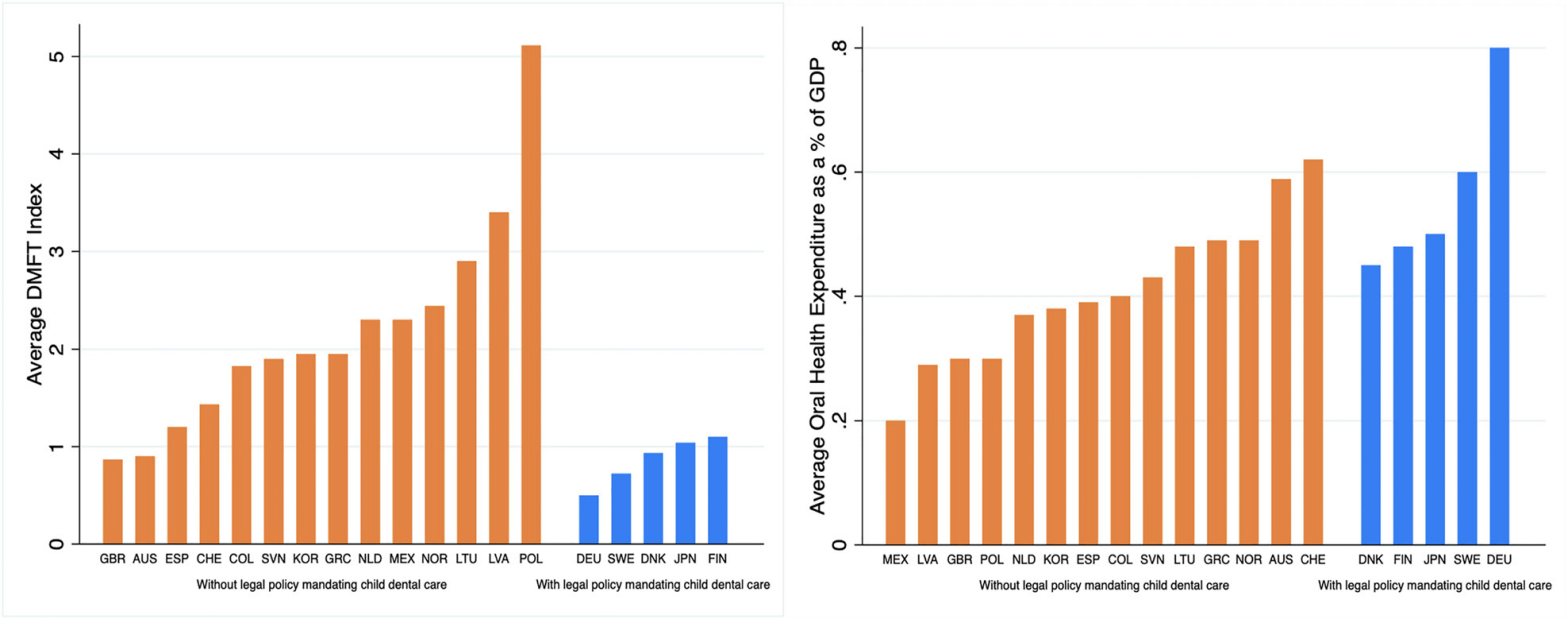
Bar graph showing country-level decayed missing and filled teeth (DMFT) index and average oral health expenditure as a percentage of GDP, categorised by oral health legal policy.

Figure 2 illustrates the relationship between the presence of legal policy for *mandatory dental services for children* and country-level DMFT index and average oral health expenditure as a percentage of GDP. Countries with legal policy for *mandatory dental services for children* all have a significantly lower average DMFT index than countries without. Countries with a legal policy to mandate oral health care for children tend to have higher oral health expenditure (as a percentage of GDP).

## Discussion

Countries’ policies on child dental care and preventive measures vary. This study found statistically significant bivariate correlations amongst legal policy that mandates dental care for children, the respective country's oral health expenditure, and DMFT index of children aged 12 to 18 years. The presence of legal policy mandating dental care for children is associated with a reduction in the respective country's DMFT index, but a slight increase in oral health expenditure.

Although the presence of legal policy mandating dental care for children is not associated with lower oral health expenditures, the benefit of the significant reduction in DMFT indexes may be worth the slightly higher spending. Most countries with legal policy mandating dental care for children are countries that were ranked with the highest efficiency indexes in a study evaluating the relationship between oral health expenditure and oral health rankings of European countries.^20^ This pattern adds to the evidence that mandating dental care through legislation increases the efficiency of the services.

Each one of the countries that has legal policies mandating dental care for children and free municipal basic and preventive services for children to prevent dental diseases also has dental services tied very closely to their schooling systems. For instance, since 1972, Denmark has implemented the Child Dental Health Care Act mandating that municipalities build dental clinics and provide basic and preventive services to children free of charge.^24^^,^^25^ Because Danish children receive compulsory education, free dental services tied to the school system are efficient.^25^

Spain and Switzerland were the only 2 countries with no policies for any category included in our study. These countries both operate under privatised oral health care models; dental care is largely excluded from mandatory health insurance and provided by private practitioners, paid directly by patients.^24^^,^^25^ In Spain, primary health care providers offer some preventive care for children, and the government provides some basic coverage to children aged 6 to 15 years. The services provided vary drastically by region and there is no national programme/oral health coverage for children. Whilst both Spain and Switzerland share relatively low DMFT index averages compared to other countries, Spain has moderate oral health expenditures, whilst Switzerland has the second highest oral health expenditure of the countries studied. It is possible that Switzerland's high oral health expenditure is due to the lack of focus on prevention and dental treatment aimed towards children.

Studies show that low-income countries are more heavily affected by dental diseases and suffer from underutilisation of available resources,^12^ likely and partially attributed to knowledge gaps. Whilst low-income countries were not included in our analysis, we reason that these countries will likely benefit from an increase in regulation of oral health policy, provided that resources are available to implement and enforce these policies.

Our study only investigated the existence of policies and not the actual scope of coverage. Future studies should further examine the laws mandating dental care, the scope of services available to children, and the details of technical guidelines/standards, allowing for examination of the effects of preventive measures on oral health outcomes and cost of care. A database that contains systematic coding of country-level oral health policies and legislations would contribute to more extensive and standardised research and our understanding on the impact of specific dental policies in different contexts as well.

## Conflict of interest

None disclosed.

## Appendix Supplementary materials

Image, application 1

## Footnotes

Supplementary material associated with this article can be found in the online version at doi:10.1016/j.identj.2023.01.007.
