# Supplementary material for: National Oral Health Policy and Financing and Dental Health Status in 19 Countries
Source: Int Dent J. 2023 Mar 21;73(3):449–55. doi: 10.1016/j.identj.2023.01.007 (PMC10213794; doi:10.1016/j.identj.2023.01.007)

**Appendix A**

DMFT Data Points Details & Sources Provided by the WHO CAPP

Documentation of each data point included in each country’s average DMFT index for this study. The table includes the year the data was taken, the age of the children, the average DMFT score of that group during the specified year as well as the collection source of the data.

| Country | Year | Age | DMFT | Source |
| --- | --- | --- | --- | --- |
| Australia | 2012 | 12 | 0.9 | The National Child Oral Health Study 2012–14 |
| Colombia | 2010 | 12 | 1.7 | Builes AMV. Saldarriaga AFS. Pino NM. Galvis MM. Colorado KC. Zuluaga YPM. Sarmiento YV. Suarez AAA. Dental caries and treatment needs in 12-year-old schoolchildren from public schools of the municipality of Rionegro (Antioquia. Colombia). 2010. Rev Fac de Odont Universidad de Antioquia. 2012; 23: 292-305. |
| Colombia | 2014 | 12 | 1.5 | IV Estudio Nacional de Salud Bucal |
| Colombia | 2014 | 15 | 2.4 | IV Estudio Nacional de Salud Bucal |
| Denmark | 2010 | 12 | 0.6 | Danish Health and Medicines Authority (National Board of Health). Dr Lene Vilstrup. chief Dental Officer. May 2013. |
| Denmark | 2011 | 12 | 0.6 | Danish Health and Medicines Authority (National Board of Health). Dr Lene Vilstrup. chief Dental Officer. May 2013. |
| Denmark | 2012 | 12 | 0.6 | Danish Health and Medicines Authority (National Board of Health). Dr Lene Vilstrup. chief Dental Officer. May 2013. |
| Denmark | 2012 | 15 | 1.3 | Danish Health and Medicines Authority (National Board of Health), Dr Lene Vilstrup, chief Dental Officer, May 2013. |
| Denmark | 2012 | 18 | 2.4 | Danish Health and Medicines Authority (National Board of Health), Dr Lene Vilstrup, chief Dental Officer, May 2013. |
| Denmark | 2014 | 12 | 0.4 | Danish Health and Medicines Authority (National Board of Health). Dr Lene Vilstrup. Chief Dental Officer. Nov 2015. |
| Denmark | 2014 | 15 | 1.1 | Danish Health and Medicines Authority (National Board of Health), Dr Lene Vilstrup, Chief Dental Officer, Nov 2015. |
| Denmark | 2014 | 18 | 2.1 | Danish Health and Medicines Authority (National Board of Health), Dr Lene Vilstrup, Chief Dental Officer, Nov 2015. |
| Denmark | 2019 | 12 | 0.3 | Tandplejeinformation OPP |
| Denmark | 2019 | 15 | 0.8 | Tandplejeinformation OPP |
| Denmark | 2019 | 18 | 1.4 | Tandplejeinformation OPP |
| Finland | 2011 | 12 | 1.3 | The Sotkanet Indicator Bank, National Institute for Health and Welfare (THL) |
| Finland | 2012 | 12 | 1.2 | The Sotkanet Indicator Bank, National Institute for Health and Welfare (THL) |
| Finland | 2013 | 12 | 1.2 | The Sotkanet Indicator Bank, National Institute for Health and Welfare (THL) |
| Finland | 2014 | 12 | 1.2 | The Sotkanet Indicator Bank, National Institute for Health and Welfare (THL) |
| Finland | 2015 | 12 | 1.2 | The Sotkanet Indicator Bank, National Institute for Health and Welfare (THL) |
| Finland | 2016 | 12 | 1.1 | The Sotkanet Indicator Bank, National Institute for Health and Welfare (THL) |
| Finland | 2017 | 12 | 0.9 | The Sotkanet Indicator Bank, National Institute for Health and Welfare (THL) |
| Finland | 2018 | 12 | 0.9 | The Sotkanet Indicator Bank, National Institute for Health and Welfare (THL) |
| Finland | 2019 | 12 | 0.9 | The Sotkanet Indicator Bank, National Institute for Health and Welfare (THL) |
| Germany | 2014 | 12 | 0.5 | Jordan AR, Micheelis W: The Fifth German Oral Health Study (DMS V). Institute of German Dentists. Deutscher Zahnaerzteverlag DÄV, Köln 2016 |
| Germany | 2014 | 12 | 0.5 | Jordan AR. Micheelis W: The Fifth German Oral Health Study (DMS V). Institute of German Dentists. Deutscher Zahnaerzteverlag DÄV. Köln 2016. |
| Greece | 2011 | 12 | 1.5 | Oulis C.J.. Tsinidou K.. Vadiakas G.. Mamai-Homata E.. Polychronopoulou A.. Athanaslouli T. (2012) Caries prevalence of 5. 12 and 15-year-old Greek children: a national pathfinder survey. Community Dental Health; 29: 29 - 32. Abstract Pubmed |
| Greece | 2011 | 12 | 2 | Oulis C.J.. Tsinidou K.. Vadiakas G.. Mamai-Homata E.. Polychronopoulou A.. Athanaslouli T. (2012) Caries prevalence of 5. 12 and 15-year-old Greek children: a national pathfinder survey. Community Dental Health; 29: 29 - 32. Abstract Pubmed |
| Greece | 2011 | 12 | 2.1 | Oulis C.J.. Tsinidou K.. Vadiakas G.. Mamai-Homata E.. Polychronopoulou A.. Athanaslouli T. (2012) Caries prevalence of 5. 12 and 15-year-old Greek children: a national pathfinder survey. Community Dental Health; 29: 29 - 32. Abstract Pubmed |
| Greece | 2011 | 12 | 2.2 | Oulis C.J.. Tsinidou K.. Vadiakas G.. Mamai-Homata E.. Polychronopoulou A.. Athanaslouli T. (2012) Caries prevalence of 5. 12 and 15-year-old Greek children: a national pathfinder survey. Community Dental Health; 29: 29 - 32. Abstract Pubmed |
| Japan | 2010 | 12 | 1.3 | National School HealthStatistics |
| Japan | 2011 | 12 | 1.4 | Survey of Dental Diseases 2011. Ministry of Health and Welfare. 2012 |
| Japan | 2013 | 12 | 1.1 | National School Health Statistics. |
| Japan | 2014 | 12 | 1 | National School Health Statistics. |
| Japan | 2015 | 12 | 0.9 | National School Health Statistics. |
| Japan | 2016 | 12 | 0.2 | Survey of Dental Diseases 2011. Ministry of Health and Welfare. 2012. |
| Japan | 2016 | 12 | 0.8 | National School Health Statistics. |
| Japan | 2016 | 14 | 0.6 | Survey of Dental Diseases 2011, Ministry of Health and Welfare, 2012 |
| Korea, Republic of | 2010 | 12 | 2.1 | Caries Control throughout Life in Asia- Conference book. International Conference. 20-22. Nov. 2013. Krabi. Thailand. Editors- Editors: Songpaisan Y.. Nawachind KM.. Ungchusak C. |
| Korea, Republic of | 2012 | 12 | 1.8 | Caries Control throughout Life in Asia- Conference book. International Conference. 20-22. Nov. 2013. Krabi. Thailand. Editors- Editors: Songpaisan Y.. Nawachind KM.. Ungchusak C. |
| Latvia | 2011 | 12 | 3.4 | Hysi D. Eaton KA. Tsakos G. Vassallo P. Amariei C and the DPH Group.Proceedings of a workshop. held in Constanta. Romania on 22 May 2014. on Oral Health of Children in the Central and Eastern European Countries in the context of the current economic crisis. BMC Oral Health 2016; 16 (Suppl 1):69:1-21 |
| Latvia | 2016 | 12 | 3.4 | Maldupa I. Uribeb S. Nizamovsa M. Senakolaa E. Caries Prevalence and Risk Factors Among 12-Year-Old Latvian Schoolchildren. Caries Res 2017;51:290–385. (Abstracts: 64th ORCA Congress) |
| Lithuania | 2014 | 18 | 2.9 | Žemaitienė M, Grigalauskienė R, Vasiliauskienė I, Saldūnaitė K, Razmienė J, Slabšinskienė E. Prevalence and severity of dental caries among 18-year-old Lithuanian adolescents. Medicina (Kaunas). 2016;52(1):54-60. Epub 2016 Jan 29. |
| Mexico | 2010 | 12 | 1.1 | Verdugo Díaz. RDJ. Llodra Calvo JC. Sánchez-Rubio Carrillo RM. Barreras Serrano A. Sánchez-Rubio Carrillo RA. Torres Arellano M E. Gómez-Llanos Juárez. H. Estudio epidemiológico de caries dental en escolares del estado de Baja California. México. 2010/2010 Epidemiological Study of Dental Caries in Schoolchildren of the Baja California State. Mexico. Universitas Odontologica. 2013; 32: 99-108. |
| Mexico | 2011 | 12 | 1.1 | Informe de Caries Dental, Encuesta Nacional de Caries y Fluorosis Dental 2011-2014 |
| Mexico | 2011 | 15 | 1.7 | Informe de Caries Dental, Encuesta Nacional de Caries y Fluorosis Dental 2011-2014 |
| Mexico | 2018 | 12 | 2.6 | Resultados del Sistema de Vigilancia Epidemiológica de Patologías Bucales SIVEPAB 2018 |
| Mexico | 2018 | 15 | 4.7 | Resultados del Sistema de Vigilancia Epidemiológica de Patologías Bucales SIVEPAB 2018 |
| Mexico | 2018 | 18 | 5.9 | Resultados del Sistema de Vigilancia Epidemiológica de Patologías Bucales SIVEPAB 2018 |
| Netherlands | 2011 | 17 | 2.3 | Schuller A.A., van Kempen I.P.E., Poorterman J.H.G., Verrips G.H.W. (2011) A study of the oral health and prevention in juveniles. |
| Norway | 2010 | 12 | 1.3 | Statistisk sentralbyrå (Statistics Norway) |
| Norway | 2010 | 18 | 4.6 | Statistisk sentralbyrå (Statistics Norway) |
| Norway | 2011 | 12 | 1.1 | Statistisk sentralbyrå (Statistics Norway) |
| Norway | 2011 | 18 | 4.3 | Statistisk sentralbyrå (Statistics Norway) |
| Norway | 2012 | 12 | 1.1 | Statistisk sentralbyrå (Statistics Norway) |
| Norway | 2012 | 18 | 4.4 | Statistisk sentralbyrå (Statistics Norway) |
| Norway | 2013 | 12 | 1 | Statistisk sentralbyrå (Statistics Norway) |
| Norway | 2013 | 18 | 4.2 | Statistisk sentralbyrå (Statistics Norway) |
| Norway | 2014 | 12 | 1 | Statistisk sentralbyrå (Statistics Norway) |
| Norway | 2014 | 18 | 4 | Statistisk sentralbyrå (Statistics Norway) |
| Norway | 2015 | 12 | 0.9 | Statistisk sentralbyrå (Statistics Norway) |
| Norway | 2015 | 18 | 3.9 | Statistisk sentralbyrå (Statistics Norway) |
| Norway | 2016 | 12 | 0.9 | Statistisk sentralbyrå (Statistics Norway) |
| Norway | 2016 | 18 | 3.7 | Statistisk sentralbyrå (Statistics Norway) |
| Norway | 2017 | 12 | 0.9 | Statistisk sentralbyrå (Statistics Norway) |
| Norway | 2017 | 18 | 3.4 | Statistisk sentralbyrå (Statistics Norway) |
| Norway | 2018 | 12 | 0.9 | Statistisk sentralbyrå (Statistics Norway) |
| Norway | 2018 | 18 | 3.3 | Statistisk sentralbyrå (Statistics Norway) |
| Norway | 2019 | 12 | 0.8 | Statistisk sentralbyrå (Statistics Norway) |
| Norway | 2019 | 15 | 1.9 | Statistisk sentralbyrå (Statistics Norway) |
| Norway | 2019 | 18 | 3.1 | Statistisk sentralbyrå (Statistics Norway) |
| Poland | 2011 | 15 | 6.1 | Personal communications Dr Urszula Kaczmarek, Oct 2016. |
| Poland | 2012 | 12 | 3.5 | Olczak-Kowalczyk D. Turska A. Gozdowski D. Kaczmarek U. Dental Caries Level and Sugar Consumption in 12-Year-Old Children from Poland. Adv Clinic Exp Med. 2016;25:545-550. ;Personal communications Dr Urszula Kaczmarek. |
| Poland | 2012 | 18 | 8 | Olczak-Kowalczyk D, Turska A, Gozdowski D, Kaczmarek U. Dental Caries Level and Sugar Consumption in 12-Year-Old Children from Poland. Adv Clinic Exp Med. 2016;25:545-550. And personal communications Dr Urszula Kaczmarek |
| Poland | 2014 | 12 | 2.8 | Bachanek T. Szatko F. Strużycka I. Małkiewicz E. Gaszyńska E. Wolańska-Klimkiewicz E. Hendzel B. Nakonieczna-Rudnicka M. Topola J. Dynowska B. Garus-Pakowska A. Godala M. Kontarska M. Kobyłecka E: Oral health monitoring. Monitoring of oral health status of Polish population in 2013-2015. Poland 2014. ISBN: 978-83-7637-317-1. [In Polish] (with the consent of Prof. Dorota Olczak-Kowalczyk. the National Cosultant in Paediatric Dentistry) |
| Poland | 2015 | 15 | 5.8 | Personal communications Dr Urszula Kaczmarek, Oct 2016. |
| Slovenia | 2013 | 12 | 1.9 | Vrbic V. Vrbic M. epidemiology of caries in 12-year-olds in Slovenia 1087-2013. Oral Health Prev Dent. 2016;14:467-473. |
| Spain | 2010 | 12 | 1.1 | Llodra Calvo JC. Encuesta de Salud Oral en España 2010. RCOE 2012;17:13-46 |
| Spain | 2010 | 15 | 1.7 | Llodra Calvo JC. Encuesta de Salud Oral en España 2010. RCOE 2012;17:13-46 |
| Spain | 2015 | 12 | 0.7 | Bravo Pérez, M., Almerich Silla, J. M., Ausina Márquez, V., Avilés Gutiérrez, P., Blanco González, J. M., Canorea Díaz, E., ... & Sainz Ruiz, C. (2016). Encuesta de salud oral en España 2015. RCOE. Revista del Consejo General de Colegios de Odontólogos y Estomatólogos de España, 2016, vol. 21, num. Sup. 1, p. 8-48. |
| Spain | 2015 | 15 | 1.3 | Bravo Pérez, M., Almerich Silla, J. M., Ausina Márquez, V., Avilés Gutiérrez, P., Blanco González, J. M., Canorea Díaz, E., ... & Sainz Ruiz, C. (2016). Encuesta de salud oral en España 2015. RCOE. Revista del Consejo General de Colegios de Odontólogos y Estomatólogos de España, 2016, vol. 21, num. Sup. 1, p. 8-48. |
| Sweden | 2010 | 12 | 0.8 | Swedish National Board of Health and Welfare. Address: Socialstyrelsen. S-106 30 Stockholm. Sweden |
| Sweden | 2011 | 12 | 0.8 | Swedish National Board of Health and Welfare. Address: Socialstyrelsen. S-106 30 Stockholm. Sweden |
| Sweden | 2013 | 12 | 0.7 | Swedish National Board of Health and Welfare. Address: Socialstyrelsen. S-106 30 Stockholm. Sweden |
| Sweden | 2014 | 12 | 0.7 | Swedish National Board of Health and Welfare. Address: Socialstyrelsen. S-106 30 Stockholm. Sweden |
| Sweden | 2015 | 12 | 0.7 | Swedish National Board of Health and Welfare. Address: Socialstyrelsen. S-106 30 Stockholm. Sweden |
| Sweden | 2016 | 12 | 0.7 | Swedish National Board of Health and Welfare. Address: Socialstyrelsen. S-106 30 Stockholm. Sweden |
| Sweden | 2017 | 12 | 0.7 | Swedish National Board of Health and Welfare. Address: Socialstyrelsen. S-106 30 Stockholm. Sweden |
| Sweden | 2018 | 12 | 0.7 | The Swedish Quality Registry for caries and periodontal disease (SKaPa) |
| Sweden | 2019 | 12 | 0.7 | The Swedish Quality Registry for caries and periodontal disease (SKaPa) |
| Switzerland | 2011 | 12 | 0.9 | Waltimo T. Menghini G. Weber C. Kulik EM. Schild S. Meyer J. Caries experience in 7-. 12-. and 15-year-old schoolchildren in the canton of Basel-Landschaft. Switzerland. from 1992 to 2011. Community Dent Oral Epidemiol. 2015 Dec 28 |
| Switzerland | 2011 | 15 | 1.7 | Waltimo T, Menghini G, Weber C, Kulik EM, Schild S, Meyer J. Caries experience in 7-, 12-, and 15-year-old schoolchildren in the canton of Basel-Landschaft, Switzerland, from 1992 to 2011. Community Dent Oral Epidemiol. 2015 Dec 28. |
| Switzerland | 2011 | 15 | 1.7 | Waltimo T, Menghini G, Weber C, Kulik EM, Schild S, Meyer J. Caries experience in 7-, 12-, and 15-year-old schoolchildren in the canton of Basel-Landschaft, Switzerland, from 1992 to 2011. Community Dent Oral Epidemiol. 2015 Dec 28. |
| United Kingdom | 2010 | 12 | 0.7 | National Dental Inspection Programme of Scotland. 2011 |
| United Kingdom | 2013 | 12 | 0.6 | National Dental Inspection Programme (NDIP), Scotland, 2013. Report of the 2013 Detailed National Dental Inspection Programme of Primary 7 children and the Basic Inspection of Primary 1 and Primary 7 children.(http://www.isdscotland.org/Health-Topics/Dental-Care/Publications/2013-10-29/2013-10-29-NDIP-Report.pdf) |
| United Kingdom | 2013 | 12 | 0.6 | National Dental Inspection Programme (NDIP). Scotland. 2013. Report of the 2013 Detailed National Dental Inspection Programme of Primary 7 children and the Basic Inspection of Primary 1 and Primary 7 children. |
| United Kingdom | 2013 | 12 | 0.8 | Children’s Dental Health Survey 2013. Report 2: Dental Disease and Damage in Children England. Wales and Northern Ireland. Health and Social Care Information Centre. 2015. |
| United Kingdom | 2013 | 12 | 0.8 | Children’s Dental Health Survey 2013. Report 2: Dental Disease and Damage in Children England. Wales and Northern Ireland. Health and Social Care Information Centre. 2015. |
| United Kingdom | 2013 | 12 | 1.3 | Children’s Dental Health Survey 2013. Report 2: Dental Disease and Damage in Children England. Wales and Northern Ireland. Health and Social Care Information Centre. 2015. |
| United Kingdom | 2013 | 12 | 1.7 | Children’s Dental Health Survey 2013. Report 2: Dental Disease and Damage in Children England. Wales and Northern Ireland. Health and Social Care Information Centre. 2015. |
| United Kingdom | 2013 | 15 | 1.3 | Children’s Dental Health Survey 2013. Report 2: Dental Disease and Damage in Children England, Wales and Northern Ireland. Health and Social Care Information Centre. 2015 |
| United Kingdom | 2013 | 15 | 1.4 | Children’s Dental Health Survey 2013. Report 2: Dental Disease and Damage in Children England, Wales and Northern Ireland. Health and Social Care Information Centre. 2015 |
| United Kingdom | 2013 | 15 | 2.2 | Children’s Dental Health Survey 2013. Report 2: Dental Disease and Damage in Children England, Wales and Northern Ireland. Health and Social Care Information Centre. 2015 |
| United Kingdom | 2013 | 15 | 3.3 | Children’s Dental Health Survey 2013. Report 2: Dental Disease and Damage in Children England, Wales and Northern Ireland. Health and Social Care Information Centre. 2015 |
| United Kingdom | 2017 | 12 | 0.5 | National Dental Inspection Programme (NDIP). Scotland. 2017. Report of the 2017 Detailed National Dental Inspection Programme of Primary 7 children and the Basic Inspection of Primary 1 and Primary 7 children. |

The Years Included in Each Country’s DMFT Average Based on Available Data

The average DMFT of each country was created for the years 2010 – 2019. However, not every country had available data for each year. The following table documents what years were available for each country.

| **Country** | **Years Included** |
| --- | --- |
| Australia | 2012 |
| Colombia | 2010, 2014 |
| Denmark | 2010, 2011, 2012, 2014, 2019 |
| Finland | 2011, 2012, 2013, 2014, 2015, 2016, 2017, 2018, 2019 |
| Germany | 2014 |
| Greece | 2011 |
| Japan | 2010, 2011, 2013, 2014, 2015, 2016 |
| Korea | 2010, 2012 |
| Latvia | 2011, 2016 |
| Lithuania | 2014 |
| Mexico | 2010, 2011, 2018 |
| Netherlands | 2011 |
| Norway | 2010, 2011, 2012, 2013, 2014, 2015, 2016, 2017, 2018, 2019 |
| Poland | 2011, 2012, 2013, 2014, 2015 |
| Slovenia | 2013 |
| Spain | 2010, 2015 |
| Sweden | 2010, 2011, 2013, 2014, 2015, 2016, 2017, 2018, 2019 |
| Switzerland | 2011 |
| United Kingdom | 2010, 2013, 2017 |

**Appendix B**

Missing Years from Oral Health Expenditure Data

The oral health expenditure data was available for the years 2010 to 2020; however, many values were missing for 2020. Thus, we excluded the limited data from 2020 and generated the mean value for the years between 2010 and 2019. The average oral health expenditure for the years 2010-2019 was calculated, except certain years were missing for the following countries:

| Country | Year(s) Excluded |
| --- | --- |
| Colombia | 2010, 2011, 2012, 2018, 2019 |
| United Kingdom | 2010, 2011, 2012 |

**Appendix C**

The Law/Act for Each Country Mandating Dental Care for Children

The name of the specific law/act mandating dental care for children for every country that one was found for.

| Country | Legal Policy Mandating Dental Care for Children |
| --- | --- |
| Denmark | Child Dental Health Care Act |
| Finland | Health Care Act |
| Germany | Section 21 Social Security Code V |
| Japan | School Health and Safety Act |
| Sweden | Public Dental Service Requirements |

A Summary of How Each Country was Categorized based on the Information Found

1. **Australia***Mandatory dental services for children (legal policy)/ Dental services through schools: No*

   *National accessible dental services for children: Yes*“The CDBS commenced operation on 1 January 2014. The program provides eligible children aged between 2-17 years access to up to $1,000 in benefits for basic dental services, with benefits capped over two consecutive calendar years. General dental services are covered and include examinations, x-rays, cleaning, fissure sealing, fillings, root canals, extractions and partial dentures. Benefits are not available for orthodontic, cosmetic dental work or services provided in a hospital. Benefits are not payable where a private health insurance benefit has been paid in respect of the dental service or if the service was provided by a disqualified practitioner.”
   <https://www.health.gov.au/resources/publications/report-on-the-fourth-review-of-the-dental-benefits-act-2008>*Guidelines/Technical Standards: No*
2. **Colombia***Mandatory dental services for children (legal policy)/ Dental services through schools: No*

   *National accessible dental services for children: No*

   *Guidelines/Technical Standards: ​​Yes
   Technical Standards for Preventive Care in Oral Health*<https://docplayer.es/9854409-Norma-tecnica-para-la-atencion-preventiva-en-salud-bucal.html>
3. **Denmark***Mandatory dental services for children (legal policy)/ Dental services through schools: Yes*
   Child Dental Health Care Act (1972)
   “In 1972, the Child Dental Health Care Act was implemented. The aim of the public dental health care for children was to prevent the onset of dental disease, to intercept disease processes and anomalies as early as possible, and to secure immediate and adequate treatment. In order to achieve this aim, it was made mandatory for all municipalities to build dental clinics and employ dental personnel to carry out the necessary procedures, containing: 1) General preventive measures, including health education; 2) Individual preventive measures; 3) Regular examinations and supervision of growth and development; 4) Full dental treatment. All dental care should be free of charge for the children.”
   <https://www.tandfonline.com/doi/pdf/10.3109/02813438509013927>
   “By law (in accordance with the Act on Child Dental Health in 1972) all children in compulsory education (from seven to 16 years of age) in Denmark were offered free dental service.”
   <https://pressbooks.pub/lacascada/chapter/chapter-1/>*National accessible dental services for children: Yes*“Oral healthcare is provided in one of two ways. For children under 18, all care is free of charge and is usually provided at school. For adults a system of government subsidies is available through private dental practitioners for most common types of treatment.”
   <https://www.omd.pt/content/uploads/2017/12/ced-manual-2015-completo.pdf><https://www.finlex.fi/en/laki/kaannokset/2010/en20101326.pdf><https://bmcoralhealth.biomedcentral.com/articles/10.1186/s12903-019-0828-z#:~:text=In%20Finland%2C%20the%20public%20sector,below%2018%20years%20of%20age>.

   *Guidelines/Technical Standards: Yes*National Clinical Guidelines
   <https://www.sundhedsstyrelsen.dk/en/English/Expertise-and-guidance/Healthcare-professionals/National-Clinical-Guidelines>
4. **Finland***Mandatory dental services for children (legal policy)/ Dental services through schools: Yes*Health Care Act (1972, 2010):
   “Local authorities shall provide within their area maternity and child health clinic services for pregnant women and for families that are expecting a child as well as for children under school age and their families. Maternity and child health clinic services include: oral health checks for children at least every other year;”

   “Local authorities shall provide school health services for pupils enrolled in educational institutions providing basic education in their area. School health services shall also cover health care during work experience placements. School health services include: oral health care for pupils, including oral health checks on at least three occasions and according to individual needs;”
   <https://www.finlex.fi/en/laki/kaannokset/2010/en20101326.pdf>*National accessible dental services for children: Yes*“In Finland, the public sector has catered for children’s dental care since the 1950s, when the school dental services started. In 1972, the Primary Health Care Act obliged municipalities to provide annual examinations and all necessary preventive and dental care free of charge to all persons below 18 years of age. Generally this is undertaken all over the country in the Public Dental Service (PDS).”
   <https://bmcoralhealth.biomedcentral.com/articles/10.1186/s12903-019-0828-z#:~:text=In%20Finland%2C%20the%20public%20sector,below%2018%20years%20of%20age>.

   *Guidelines/Technical Standards: No*
5. **Germany***Mandatory dental services for children (legal policy)/ Dental services through schools: Yes

   Section 21 Social Security Code V
   “Paragraph 21 of SGB V states the following: Prevention of dental diseases (group prophylaxis):
   (1) The health insurance companies, in cooperation with the dentists and the offices responsible for dental health care in the federal states, have to promote and promote joint and uniform measures for the detection and prevention of dental diseases of their insured persons who have not yet reached the age of twelve, without prejudice to the tasks of others share in the cost of implementation. You have to work towards comprehensive measures. In schools and facilities for the disabled, where the average risk of caries among students is disproportionately high, the measures are carried out up to the age of 16.* The measures should primarily be carried out in groups, especially in kindergartens and schools; they should focus in particular on examining the oral cavity, assessing the status of the teeth, hardening of the enamel, nutritional advice and oral hygiene. Specific programs should be developed for children with a particularly high risk of caries.”*[*https://www-daj-de.translate.goog/Gesetzliche-Grundlage.43.0.html?_x_tr_sl=de&_x_tr_tl=en&_x_tr_hl=en&_x_tr_pto=sc*](https://www-daj-de.translate.goog/Gesetzliche-Grundlage.43.0.html?_x_tr_sl=de&_x_tr_tl=en&_x_tr_hl=en&_x_tr_pto=sc)**“**So-called group prophylaxis is provided in schools and covered jointly by the sickness funds, the federal dental chambers and associations of public dentists as well as federal state governments.”
   <https://eurohealthobservatory.who.int/publications/i/oral-health-care-in-europe-financing-access-and-provision>[*https://www-daj-de.translate.goog/gruppenprophylaxe.27.0.html?_x_tr_sl=de&_x_tr_tl=en&_x_tr_hl=en&_x_tr_pto=sc*](https://www-daj-de.translate.goog/gruppenprophylaxe.27.0.html?_x_tr_sl=de&_x_tr_tl=en&_x_tr_hl=en&_x_tr_pto=sc)**“***At federal level they are organised in Regional Working Groups for Children's Dentistry and Dental Hygiene and are responsible for the implementation of group prevention, which was outlined in federal laws for the health system in 2000.”*[*https://www.nature.com/articles/sj.bdj.2015.95*](https://www.nature.com/articles/sj.bdj.2015.95)*National accessible dental services for children: Yes*
   “The sick funds offer full compensation for all medically necessary conservative and surgical dental treatment as well as necessary orthodontist care for persons aged less than 18. Persons under 18 are also entitled to receive certain prophylactic treatments free of charge.”
   <https://www.omd.pt/content/uploads/2017/12/ced-manual-2015-completo.pdf><https://journals.sagepub.com/doi/full/10.1177/0022034514565648>*Guidelines/Technical Standards: No*
6. **Greece***Mandatory dental services for children (legal policy)/ Dental services through schools: No

   National accessible dental services for children: Yes*
   “The NHS provides free healthcare to all. NHS health centers emphasize more on preventive and other simple treatments to children under the age of 18, without excluding the rest of the population.”
   <https://www.omd.pt/content/uploads/2017/12/ced-manual-2015-completo.pdf>*Guidelines/Technical Standards: No*
7. **Japan***Mandatory dental services for children (legal policy)/ Dental services through schools: Yes*“In Japan, every public primary, junior, and senior high school has an appointed school dentist. In 2014 the total number of school dentists holding such positions was 44,600. The school dentist is responsible for the performance of school-based oral health activities, usually in a part-time capacity, because s/he may work also as a dental practitioner in the area. The roles of school dentists are described in the “School Health and Safety Act” and include the conduct of an oral health examination at least once a year on each child at school, and contributing to implementing the school’s oral health education.”
   <https://www.ncbi.nlm.nih.gov/pmc/articles/PMC6163272/#:~:text=Japan%20introduced%20a%20universal%20health,is%20applied%20throughout%20the%20nation>.
    *National accessible dental services for children: Yes*“Japan introduced a universal health insurance system for the entire population in 1961. It covers almost all medical and dental treatment and pharmacy care required by the population. People can receive treatment at a relatively low cost, and the same fee is applied throughout the nation.”
   [*https://www.ncbi.nlm.nih.gov/pmc/articles/PMC6163272/#:~:text=Japan%20introduced%20a%20universal%20health,is%20applied%20throughout%20the%20nation*](https://www.ncbi.nlm.nih.gov/pmc/articles/PMC6163272/#:~:text=Japan%20introduced%20a%20universal%20health,is%20applied%20throughout%20the%20nation)*.

   Guidelines/Technical Standards: Yes*Caries Treatment Guidelines by the Japanese Society of Conservative Dentistry
   [*http://www.hozon.or.jp/member/publication/guideline/*](http://www.hozon.or.jp/member/publication/guideline/)
8. **Korea***Mandatory dental services for children (legal policy)/ Dental services through schools: No

   National accessible dental services for children: Yes*Preventive treatments covered by NHIS services
   <https://www.ncbi.nlm.nih.gov/pmc/articles/PMC8067770/><https://www.mdpi.com/1660-4601/17/17/6417/htm><https://bmcoralhealth.biomedcentral.com/articles/10.1186/s12903-020-01201-8>*Guidelines/Technical Standards: No*
9. **Latvia***Mandatory dental services for children (legal policy)/ Dental services through schools: No

   National accessible dental services for children: Yes*
   “Subsequently, care for adults is privately financed, and publicly financed through the the National Health Service (NHS) for children up to the age of 18 (with the exception of orthodontic treatment).”
   <https://www.omd.pt/content/uploads/2017/12/ced-manual-2015-completo.pdf>*Guidelines/Technical Standards: No*
10. **Lithuania***Mandatory dental services for children (legal policy)/ Dental services through schools: No*
    *National accessible dental services for children: Yes*
    “Public oral health care is free of charge, for children and teenagers under the age of 18 years, and prosthodontic care for pensioners and the disabled.”
    <https://www.omd.pt/content/uploads/2017/12/ced-manual-2015-completo.pdf>*Guidelines/Technical Standards: No*
11. **Mexico***Mandatory dental services for children (legal policy)/ Dental services through schools: No

    National accessible dental services for children: No*“In Mexico, more than half of the population has all its health care costs, including dental care, met through the social security system. Another 20 per cent of the population is covered through a publicly-subsidised voluntary health programme, Seguro Popular, while a very small percentage purchase private cover. In principle, social security covers all types of medical services, including dentistry, but in practice what is offered is limited by budget constraints.”
    <https://www.aph.gov.au/About_Parliament/Parliamentary_Departments/Parliamentary_Library/pubs/BN/2011-2012/DentalSchemes><https://www.cndh.org.mx/sites/default/files/doc/Programas/VIH/Leyes%20y%20normas%20y%20reglamentos/Norma%20Oficial%20Mexicana/NOM-013-SSA2-1994%20Prevenci%C3%B3n%20y%20control%20de%20enfermedades%20bucales.pdf><https://www.gob.mx/cms/uploads/attachment/file/400143/Medina_Solis_-_Pol_ticas_de_salud_bucal_en_M_xico__disminuir_las_principales_enfermedades.pdf><http://www.cenaprece.salud.gob.mx/programas/interior/saludbucal/descargas/pdf/sbpye_completo2.pdf>*Guidelines/Technical Standards: Yes*“The ministry of health has released many Clinical Practice Guidelines one of which is the "Prevention and diagnosis of dental caries in patients aged 6 to 16 years."”
    <https://www.odontoprev.com.mx/wp-content/uploads/2021/03/rr_14.pdf>
12. **Netherlands***Mandatory dental services for children (legal policy)/ Dental services through schools: No*

    *National accessible dental services for children: Yes*
    “For young residents, up to and including 17 years of age, the basic insurance covers the costs of preventive and curative (primary) oral health care.”
    <https://www.researchgate.net/publication/342799428_Developments_in_oral_health_care_in_the_Netherlands_between_1995_and_2018>*Guidelines/Technical Standards: Yes*With the launching of the Institute of Knowledge Translation in Oral Care (KIMO), clinical guideline development has occurred
    <https://www.ntvt.nl/tijdschrift/editie/artikel/t/evidencebased-klinische-praktijkrichtlijnen-in-de-mondzorg-2-proces-en-inhoud-van-evidencebased-richtlijnontwikkeling><https://www.hetkimo.nl/richtlijnen/mondzorg-voor-jeugdigen-preventie-en-behandeling-van-caries-2020/introductie/>
13. **Norway***Mandatory dental services for children (legal policy)/ Dental services through schools: No*

    *National accessible dental services for children: Yes*“The Public Dental Health Service (PDHS) is country-wide and is organised and funded by the counties. Approximately 32.5% of all active dentists work full-time in the public sector, the remainder working in private practice. The PDHS provides dental care to priority groups and in geographic areas with few private practitioners, to non-priority adults. The five groups, in order of priority, are: children and juveniles 0-18 years the mentally handicapped people who due to long term illness are under care in institutions or at home for longer than 3 months (these groups can also receive domiciliary care) young people 19-20 years of age other groups defined by the county or the government, inter alia imprisoned persons and drug and alcoholic addicts in a rehabilitation program. Annually approximately 64% of the population in the priority groups receive screening and/or treatment (2012) and about 10% of the non-priority group adults also receive their care from the PDHS.”
    <https://www.omd.pt/content/uploads/2017/12/ced-manual-2015-completo.pdf>*Guidelines/Technical Standards: No*
14. **Poland***Mandatory dental services for children (legal policy)/ Dental services through schools: No

    National accessible dental services for children: Yes*Public compulsory health insurance
    “Subject to the Act, persons insured are entitled to the basic dental services, normally performed by a dental surgeon, as well as dental materials specified by the Minister of Health in a regulation. Children and young people under 18 years, as well as women who are pregnant and in the post-natal period (up to 42 days after childbirth) are entitled to additional services by a dental surgeon, taking into account the specific dental needs of this section of population. An insured person is entitled to a dental examination, or periodical examination, once a year. Children and young people are entitled to an additional periodical examination and a wider range of services.”
    <https://www.omd.pt/content/uploads/2017/12/ced-manual-2015-completo.pdf>*Guidelines/Technical Standards: No*
15. **Slovenia***Mandatory dental services for children (legal policy)/ Dental services through schools: No

    National accessible dental services for children: Yes*“Public compulsory health insurance provides dental cover for all patients of 0 to 18 years of age, all removable and fixed appliances, and for adults, surgical items, some basic prosthodontic treatments, periodontal and conservative treatment such as fillings and endodontics.”
    <https://www.omd.pt/content/uploads/2017/12/ced-manual-2015-completo.pdf>*Guidelines/Technical Standards: No*
16. **Spain***Mandatory dental services for children (legal policy)/ Dental services through schools: No

    National accessible dental services for children: No
    Not very comprehensively:* “Very little oral health coverage for adults but free basic services nationally for children under the Spanish National Health System since 2008 (Servicio Nacional de Salud - SNS)”
    <https://www.researchgate.net/publication/286612737_The_healthcare_system_and_the_provision_of_oral_healthcare_in_European_Union_member_states_Part_2_Spain><https://eurohealthobservatory.who.int/publications/i/oral-health-care-in-europe-financing-access-and-provision>*Guidelines/Technical Standards: No*
17. **Sweden***Mandatory dental services for children (legal policy)/ Dental services through schools: Yes*“Care is provided on a regular basis and is individually targeted. Approximately 95% of children and adolescents have contact with dental care over a two year period. Additionally, preventive dental care is provided to children in schools or child healthcare centres in terms of health promotion, information or offering preventive fluoride treatments.”
    <https://www.omd.pt/content/uploads/2017/12/ced-manual-2015-completo.pdf>*National accessible dental services for children: Yes*“Most health care in Sweden, including dentistry, is financed through a national social insurance system. A resident of Sweden must be registered with a social insurance office when they reach the age of 16.  Most dental care is provided in one of two ways: the Public Dental Service which provides free dental care to children up to the age of 19, or a subsidized dental scheme for adults who are not entitled to free care.”
    <https://www.aph.gov.au/About_Parliament/Parliamentary_Departments/Parliamentary_Library/pubs/BN/2011-2012/DentalSchemes>*Guidelines/Technical Standards: No*
18. **Switzerland***Mandatory dental services for children (legal policy)/ Dental services through schools: No

    National accessible dental services for children: No*According to the EU Manual of Dental Practice 2015 Apart from a minority of dentists employed by hospitals or the school dental service, most oral healthcare is provided by independent private practitioners and paid for directly by individual patients.
    [*https://www.omd.pt/content/uploads/2017/12/ced-manual-2015-completo.pdf*](https://www.omd.pt/content/uploads/2017/12/ced-manual-2015-completo.pdf)Considering to introduce a mandatory/compulsory dental care insurance was supposed to be voted on in 2018 - don't think it passed
    <https://www.sciencedirect.com/science/article/pii/S0168851017300994>*Guidelines/Technical Standards: No*
19. **United Kingdom***Mandatory dental services for children (legal policy)/ Dental services through schools: No

    National accessible dental services for children: Yes*
    “Since 1948 dental care has been included under the National Health Service (NHS). The NHS is largely funded through general taxation. Most patients contribute to the cost of GDS dental care through co-payments. Specific groups receive general dental care without any patient charge, for example children under 18 years old, pregnant or nursing mothers, those on welfare benefits, and those under 19 years old who are also in full-time education.”
    [*https://www.researchgate.net/profile/Daniela-Garbin/publication/275889100_A_comparative_analysis_of_oral_health_care_systems_in_the_United_States_United_Kingdom_France_Canada_and_Brazil/links/5552a85e08ae980ca606c258/A-comparative-analysis-of-oral-health-care-systems-in-the-United-States-United-Kingdom-France-Canada-and-Brazil.pdf*](https://www.researchgate.net/profile/Daniela-Garbin/publication/275889100_A_comparative_analysis_of_oral_health_care_systems_in_the_United_States_United_Kingdom_France_Canada_and_Brazil/links/5552a85e08ae980ca606c258/A-comparative-analysis-of-oral-health-care-systems-in-the-United-States-United-Kingdom-France-Canada-and-Brazil.pdf)*Guidelines/Technical Standards: Yes*

*https://www.nice.org.uk/guidance/ng30*

**Appendix D**

A sensitivity analysis excluding Poland shows that Poland does not significantly change the findings of the study.

|  | **Average DMFT index** | **Average oral health expenditure as a % of GDP** | **Mandatory Dental Services for Children** | **Available Dental Services for Children** | **Technical Standards/Guidelines** |
| --- | --- | --- | --- | --- | --- |
| Average DMFT index | 1.00 |  |  |  |  |
| Average oral health expenditure as a % of GDP | -3.15* | 1.00 |  |  |  |
| Mandatory Dental Services for Children | -1.09* | 0.15* | 1.00 |  |  |
| Available Dental Services for Children | -0.05 | 0.07 | 0.36 | 1.00 |  |
| Technical Standards/Guidelines | -0.16 | -0.13 | 0.08 | -0.17 | 1.00 |

*p<0.05


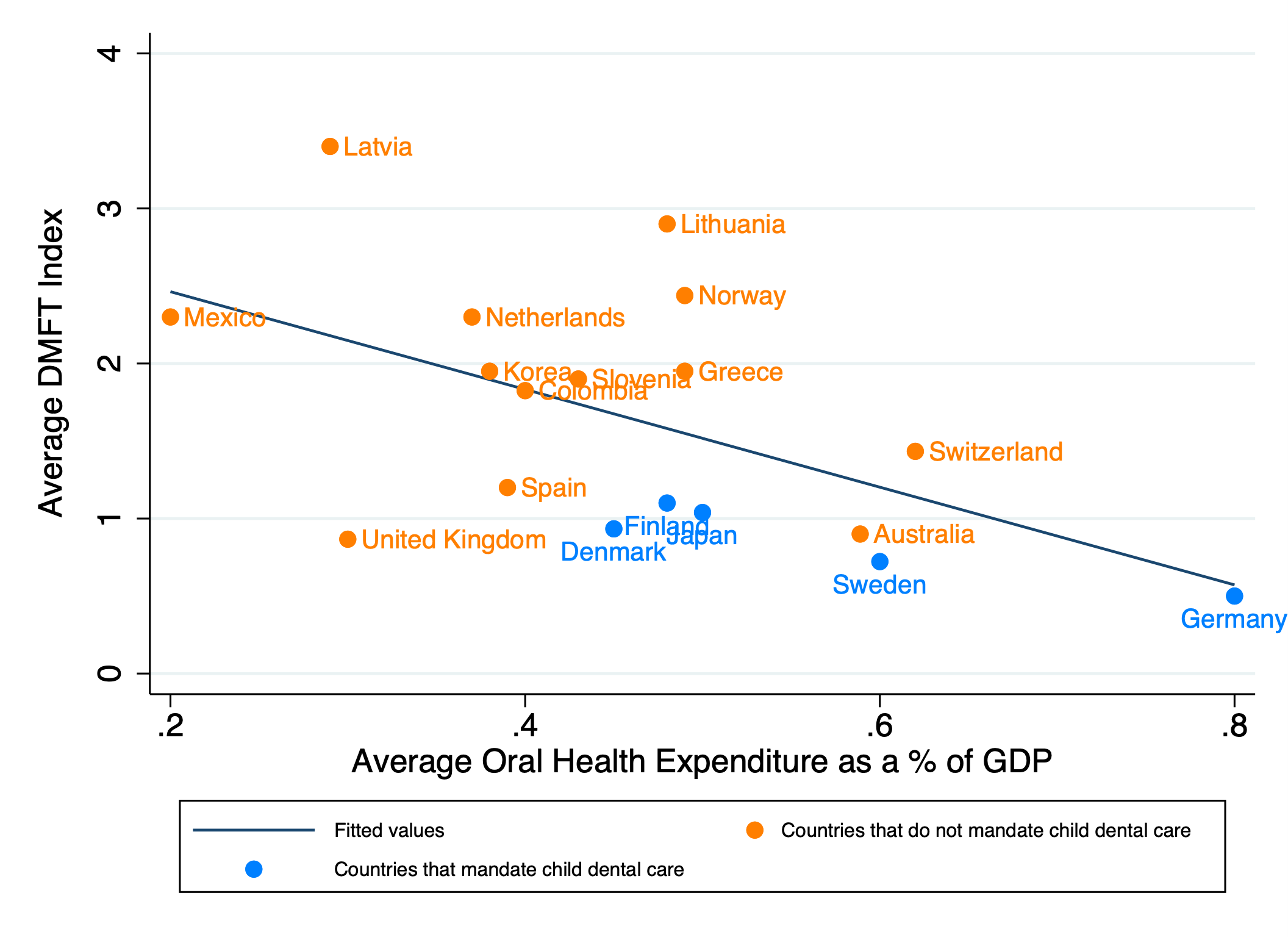

Supplement: Supplementary file 1 [file mmc1.docx]
